# A reliable and validated LC-MS/MS method for the simultaneous quantification of 4 cannabinoids in 40 consumer products

**DOI:** 10.1371/journal.pone.0196396

**Published:** 2018-05-02

**Authors:** Qingfang Meng, Beth Buchanan, Jonathan Zuccolo, Mathieu-Marc Poulin, Joseph Gabriele, David Charles Baranowski

**Affiliations:** Research and Development, Delivra Corp., Charlottetown, PE, Canada; National Institutes of Health, UNITED STATES

## Abstract

In the past 50 years, *Cannabis sativa* (*C*. *sativa*) has gone from a substance essentially prohibited worldwide to one that is gaining acceptance both culturally and legally in many countries for medicinal and recreational use. As additional jurisdictions legalize *Cannabis* products and the variety and complexity of these products surpass the classical dried plant material, appropriate methods for measuring the biologically active constituents is paramount to ensure safety and regulatory compliance. While there are numerous active compounds in *C*. *sativa* the primary cannabinoids of regulatory and safety concern are (-)-Δ⁹-tetrahydrocannabinol (THC), cannabidiol (CBD), and their respective acidic forms THCA-A and CBDA. Using the US Food and Drug Administration (FDA) bioanalytical method validation guidelines we developed a sensitive, selective, and accurate method for the simultaneous analysis CBD, CBDA, THC, and THCA-A in oils and THC & CBD in more complex matrices. This HPLC-MS/MS method was simple and reliable using standard sample dilution and homogenization, an isocratic chromatographic separation, and a triple quadrupole mass spectrometer. The lower limit of quantification (LLOQ) for analytes was 0.195 ng/mL over a 0.195–50.0 ng/mL range of quantification with a coefficient of correlation of >0.99. Average intra-day and inter-day accuracies were 94.2–112.7% and 97.2–110.9%, respectively. This method was used to quantify CBD, CBDA, THC, and THCA-A in 40 commercial hemp products representing a variety of matrices including oils, plant materials, and creams/cosmetics. All products tested met the federal regulatory restrictions on THC content in Canada (<10 μg/g) except two, with concentrations of 337 and 10.01 μg/g. With respect to CBD, the majority of analyzed products contained low CBD levels and a CBD: CBDA ratio of <1.0. In contrast, one product contained 8,410 μg/g CBD and a CBD: CBDA ratio of >1,000 (an oil-based product). Overall, the method proved amenable to the analysis of various commercial products including oils, creams, and plant material and may be diagnostically indicative of adulteration with non-hemp *C*. *sativa*, specialized hemp cultivars, or unique manufacturing methods.

## Introduction

*Cannabis sativa* is one of three generally recognized plant species of *Cannabis* [1]. *C*. *sativa* has been used for industrial textiles, food production (hemp), medicinal, and illicit psychoactive properties (marihuana) for several thousand years [2]. The biological potential of the plant has been investigated for the treatment of pain, glaucoma, nausea, asthma, depression, insomnia and neuralgia [3,4], multiple sclerosis [5], and inflammatory diseases [6,7], epilepsy [8], and movement disorders [9]. Not until the mid-20^th^ century were the cannabinoids responsible for the biological effects of *C*. *sativa* first identified [10,11].

*C*. *sativa* contains a family of approximately 60 structurally similar cannabinoids [12], however the majority of research to date has focused upon the psychoactive Δ^9^-tetrahydrocannabinol (THC) and the structurally similar non-psychoactive cannabidiol (CBD). THC is a ligand for cannabinoid receptor-1 and -2 (CB1 and CB2), which regulate a variety of basic physiological processes such as appetite, mood, memory, and inflammation [13]. As such, activation of CB1 and CB2 can yield broad neurological manifestations that are further complicated by the dissimilar molecular effects of THC or CBD [14]. Specifically, THC is a partial agonist of CB1 and CB2 whereas CBD is a negative allosteric modulator and so the overall physiological effect of *C*. *sativa* is often related to both THC and CBD content [15,16]. While THC and CBD are the most relevant cannabinoids to mammalian biology, *C*. *sativa* produces both in their inactive acidic forms [17,18].

The acidic forms of CBD and THC are cannabidiolic acid (CBDA) and Δ^9^-tetrahydrocannabinolic acid A (THCA-A), respectively. CBDA and THCA are psychologically inactive precursors that may be converted to CBD and THC via decarboxylation [19]. This conversion is promoted by heat, however the extent of conversion is dependant on the heating method [20,21]. Therefore, analytical methods that include thermal sample manipulations (e.g. gas chromatography) require chemical derivatization to evaluate CBDA and THCA-A independently of CBD and THC [22]. This has given rise to numerous liquid chromatography-based methods to evaluate both acidic and non-acidic forms.

LC-MS/MS test development has been prolific in THC forensic analysis of hair, blood, urine, and sweat [23–26]. In contrast, the application of LC-MS/MS method to the complex and diverse matrices often encountered in the food and supplements marketplace is not strongly established. Citti and colleagues developed and tested a liquid chromatography and ultraviolet spectroscopy (LC-UV) method to evaluate hemp seed oils for multiple cannabinoids [27]. However, the low sensitivity of UV spectroscopy and lack of specificity detracts from its broad applicability in complex samples. Similarly, Carcieri and colleagues developed an LC mass spectrometry (LC-MS/MS) method to test inter-lot variability in medicinal preparations of olive oil-based formulations containing *Cannabis* [28]. This investigation identified high variability in THC and CBD between lots, however, the described sample LLOQ of 100 μg/mL is insufficient to verify if products conform to hemp regulations (10 μg/g). Yang and colleagues investigated three brands of consumer-grade hemp seeds using four different procedures to extract phytocannabinoids, and quantified total THC and CBD [29]. In almost all cases, THC concentrations were reported as higher than the legal limit [30]. Given the hypothesized absence of a cannabinoid biosynthetic pathway within the seeds [31,32], the elevated THC levels observed likely arise from remaining husks or contamination by other organs. This method was specific to seeds and did not account for the complex matrices encountered in consumer goods.

Here, a sensitive, simple, and reliable method for the simultaneous quantification of THC, CBD, THCA-A and CBDA in a variety of finished commercial products is presented. The LC-MS/MS method was validated and applied to 40 commercially available hemp products including solids, oils, creams, and capsules. To our knowledge, this is the first report of a method that quantifies these primary cannabinoids in a variety of materials and is amenable to safety, quality, stability, and consistency testing.

## Experimental

### Chemicals, reagents, and test articles

Mass-validated reference standards for cannabidiol (CBD), (-)-Δ^9^-tetrahydrocannabinol (THC), cannabidiol-D_3_ (CBD-d3), (-)-Δ^9^-tetrahydrocannabinol-D_3_ (THC-d3), cannabidiolic acid (CBDA), Δ^9^-tetrahydrocannabinolic acid A (THCA-A), and (±)-11-nor-9-carboxy-Δ^9^-THC-D_3_ (THCCOOH-d3) were purchased from Sigma Aldrich (Cerilliant).

HPLC grade solvents were purchased from Caledon (acetonitrile 190 and water) and Millipore (omnisolv methanol). Formic acid was purchased from Sigma Aldrich.

Forty test articles were purchased online or in-store for analysis. They were all labelled as hemp-containing, and were offered in the form of oils, cosmetics, food products, supplements, and tinctures. The test articles were blinded and stored at 4°C upon arrival.

### Instrumentation

High performance liquid chromatography- tandem mass spectrometry was carried out on 5500 QTRAP Mass spectrometer (ABSciex, Concord, Canada) with a TurboV source, equipped with Agilent 1260 (Agilent Technologies, Santa Clara CA, USA) HPLC. Other equipment included Biofuge Fresco Heraeus centrifuge, Mettler Toledo analytical balance and mini vortexer.

### Preparation of standard solutions

A 100 ng/mL standard solution of CBD, THC, CBDA, THCA-A was prepared by dilution of stock solutions of CBD, THC (1 mg/mL in MeOH, thawed at room temperature from -20°C storage), CBDA, and THCA-A (1 mg/mL in MeOH, thawed at room temperature from -80°C storage) with dilution solvent (0.005% formic acid, 5% water, 95% methanol).

A 10 ng/mL internal standard (IS) solution consisting of CBD-d3, THC-d3 and THCCOOH-d3 was prepared from stock solutions of CBD-d3 (100 μg/mL in MeOH, thawed at RT from -20°C storage), THC-d3 (100 μg/mL in MeOH, thawed at RT from -20°C storage) and THCCOOH-d3 (1 mg/mL in MeOH, thawed at RT from -20°C storage) and dilution solvent. THC-d3 and CBD-d3 were used as internal standards for THC and CBD respectively. THCCOOH-d3 was used as the internal standard for both CBDA and THCA-A.

### Calibration curve and quality control sample preparation

A solution of 50 ng/mL of each of CBD, THC, CBDA, THCA-A and 10 ng/mL of CBD-d3, THC-d3 and THCCOOH-d3 was serial diluted with an equal volume of a solution of 10 ng/mL of CBD-d3, THC-d3 and THCCOOH-d3 to give a calibration curve with concentrations of CBD, THC, CBDA, THCA-A of 50, 25, 12.5, 6.25, 3.12, 1.56, 0.78, 0.39 and 0.19 ng/mL and a constant concentration of CBD-d3, THC-d3, and THCCOOH-d3 (10 ng/mL each). The peak area ratio of the analytes to their corresponding IS vs. concentration of analytes was fit with a weighted quadratic curve or linear curve using Analyst software 1.6.2. Quality control samples were prepared from olive oil at concentrations of 0.5, 5, 50 and 200 ng/mg for every batch of commercial products test.

### Sample preparation

The test article (oil, plant material, or cream) to be analyzed was weighed into scintillation vials in triplicate and then enough extraction solvent (10 ng/mL CBD-d3, THC-d3, and THCCOOH-d3 in methanol with 0.005% formic acid and 5% water) was added to make a 1 mg/mL solution of the test article (If the sample was chunky, such as plant material the sample was ground and homogenized using a mortar and pestle prior to weighing). The samples were then sonicated for 10 minutes at room temperature and centrifuged at 11,000 rpm for 10 minutes.

### HPLC- MS/MS conditions

Chromatographic separation was performed on an Agilent Eclipse Plus 95 Ȧ C18 column (4.6 x 100 mm, 3.5 μm particle size, Agilent 959961–902) with guard column using an isocratic mobile phase of water (0.1% formic acid): acetonitrile (0.1% formic acid) 10:90 at a flow rate of 0.5 mL/min for 11 min. The first 2 minutes was sent to the waste. The column temperature was 40°C, the autosampler temperature was maintained at 4°C and the injection volume was 20 μL. A 5500 QTRAP from ABSciex equipped with an electrospray ionization (ESI) was used in negative ion mode for detection of CBDA, THCA-A and THCCOOH-d3 and in positive ion mode for CBD, THC, CBD-d3, and THC-d3 with multiple reaction monitoring (MRM) for quantitative analysis. Nitrogen was used as the collision gas and the curtain gas.

In experiment 1 (negative mode), the curtain gas was 30.00 psi, the collision gas was MED, the ion spray voltage was -4500 V, the temperature was 600°C, and gas sources 1 and 2 were 50 and 70 psi respectively. The declustering potential was -155 V, the entrance potential was -10.00 V. Quantification was performed using the transitions m/z 357.0 → 339.0 (CE = -29 V, 100 msec, CXP = -15 V), m/z 357.0 → 313.0 (CE = -34 V, 100 msec, CXP = -7 V), and m/z 346.2 → 302.2, (CE = -22 V, 100 msec, CXP = -15 V) for CBDA, THCA-A, and THCCOOH-d3 with retention times 3.9, 8.5, and 3.45 minutes. The 2nd transitions 357.0 → 179.0 (CE = -30 V, 100 msec, CXP = -15 V), m/z 357.0 → 245.0 (CE = -43 V, 100 msec, CXP = -5 V) and m/z 346.2 → 248.1, (CE = -35 V, 100 msec, CXP = -15 V) of each analyte were used to confirm identity of CBDA, THCA-A, and THCCOOH-d3 respectively.

In experiment 2 (positive mode), the curtain gas was 30.00 psi, the collision gas was MED, the ion spray voltage was 4500 V, the temperature was 600°C, and gas sources 1 and 2 were 50 and 70 psi respectively. The de-clustering potential was 100 V, the entrance potential was 10.00 V, and the cell exit potential was 15.00 V. Quantification was performed using the transition m/z 315.0 → 193.0 (CE = 30 V, 100 msec) for both CBD (retention time—4.2 minutes) and THC (retention time—6.8 minutes), and 318.0 → 196.0 (CE = 30 V, 100 msec) for both CBD-d3 (retention time—4.2 minutes) THC-d3 (retention time—6.8 minutes). The 2nd transition 315.0 → 259.0 (CE = 30 V, 100 msec) was used to identification of CBD or THC.

### Method validation

This method was validated following the FDA Bioanalytical Method Validation Guidelines for Industry [33] and Health Canada [34]. Selectivity, limit of detection and quantification, linearity, precision, accuracy, recovery, matrix effect and essential stability were assessed. Full validation was performed using olive oil as the matrix. Partial validation for dried plant material and cream matrices were performed to evaluate accuracy, precision, recovery, and matrix effect.

### Selectivity and lower limit of quantification

To test the selectivity of this method, 8 sources of matrix were analyzed for interference including sunflower oil, coconut oil, grape seed oil, almond oil, avocado oil, two brands of olive oil and a topical cream DelivraSR. Each matrix was extracted in triplicate and the observed peak areas at the appropriate retention times in the chromatograph were compared to the LLOQ of each analyte and internal standard. Peak areas of blank matrices were required to be < 1/5 the LLOQ at the analyte retention time. Lower limit of quantification was defined as the lowest concentration that produced a peak area 5 times the blank solvent peak area, had an accuracy within 20% of the nominal value, and a precision of no more than 20% CV. LLOD was the lowest concentration that produced a peak area > 3 times blank solvent peak area.

### Calibration curve, precision, and accuracy

A calibration curve and quality control samples were included in each batch of test articles. The ratio of analyte peak area to internal standard peak area was plotted against nominal concentration. The calibration curves of CBD and THC were fit with a weighted (1/concentration) linear equation, while the calibration curves of CBDA and THCA-A were better fit with a weighted (1/concentration) quadratic equation. A total of 5 replicate dope quality control samples at each of 4 different concentrations (0.5, 5, 50 and 200 ng/mg) were extracted and tested on 3 days. Inter-day and intra-day precision and accuracy of quality control samples were calculated with the requirement that the mean of each concentration must be within 15% of the nominal value, and must have a precision not exceeding 15% CV.

### Matrix effects and recovery

Matrix effects were measured by comparing the peak area of the blank matrix extract spiked with standards to standards of the same concentration in solvent. Extraction recovery was measured by comparing the peak area of blank matrix extract spiked with standards before extraction procedure and after extraction procedure.

### Stability

Chemical stability of all analytes was evaluated under sample handling and storage conditions using five replicates of each of 0.5, 5, 50 and 200 ng/mL quality control samples. Benchtop stability was evaluated at room temperature in matrix-containing extraction solvent over a period of 12 hours, and peak area was compared with that of freshly prepared samples. Stability of processed samples was evaluated by re-injection of processed quality control samples after storage at 4°C for 3 days. Processed sample stability was calculated as the ratio of the peak area of second injection to first injection.

All the hemp products were stored at 4°C upon receipt. The product expiration dates were ≥ 1 year for most of the hemp products, and all the products were analyzed prior to their expiration date.

### Test sample analysis

Each test article was analyzed in triplicate using the validated method, accompanied by a calibration curve and quality control samples (0.5, 5, 50, and 200 ng/mL). If more than two thirds (67%) of quality control samples at 4 concentrations results were within 15% of their respective nominal (theoretical) values this resulting batch was accepted, otherwise the resulting batch was rejected. One test article in each batch was randomly selected for reanalysis to assess the reproducibility of method, two thirds (67%) of which could not differ by more than 20% between batches.

## Results and discussion

### Method development

**Fig 1:**
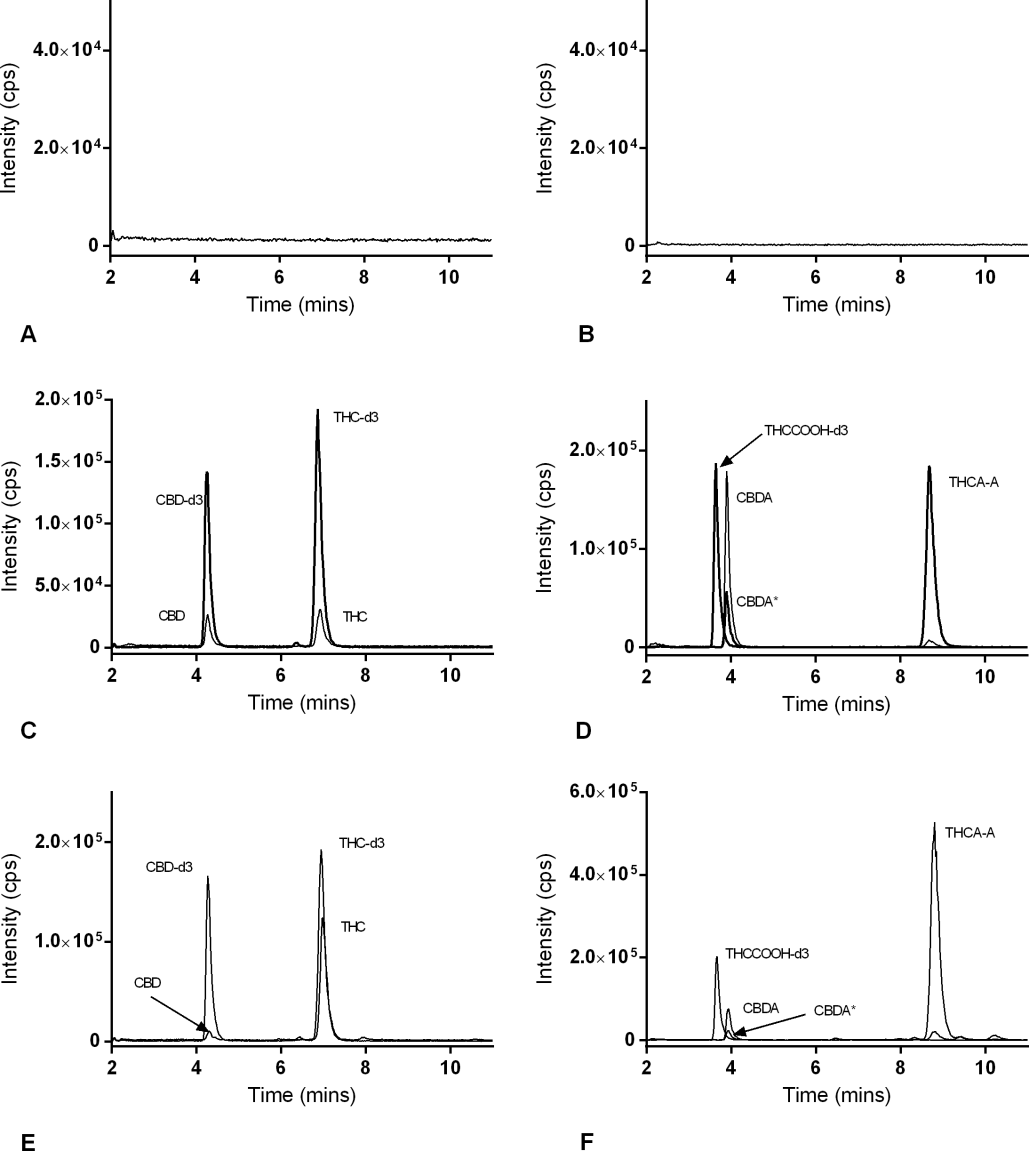
LC-MS/MS profiles of cannabinoids in matrix. Chromatograms of blank matrix (olive oil) detected in (A) positive mode, (B) negative mode, blank matrix spiked with 2 ng/mL CBD, THC, CBDA and THCA-A and 10 ng/mL CBD-d3, THC-d3 and THCCOOH-d3 detected in (C) positive mode and (D) negative mode and test article # 32 analyzed in (E) positive mode and (F) negative mode. Asterisk denotes a small peak (CBDA*) with the same MRM pair of THCA-A due to concurrent fragmentation (D and F).

An accurate and robust analytical method has been developed for the quantification of 4 cannabinoids relevant to the health and safety of *C*. *sativa* users. The HPLC-MS/MS method has the benefit of being extremely sensitive, and accommodates 4 analytes at once. All MS/MS parameters were optimized using standard solutions of single analytes. The isomers: CBD and THC were monitored in positive ion mode using an identical MRM transition (315.0 → 193.0) and the compounds were distinguished by retention time (Fig 1C). A second transition was used for qualification of each analyte, and deuterated standards were used as internal standards. Negative ion mode was more efficient for the ionization of CBDA and THCA-A. Deuterated analogues of CBDA and THCA-A were not commercially available, thus THCCOOH-d3 was chosen for an internal standard for both CBDA and THCA-A, also monitored in negative ion mode. CBDA and THCA-A were monitored using a common parent ion in their MRM transitions (357.0 → 339.0, and 357.0 → 313.0) but differing fragment ions. A small percentage of CBDA fragmented to yield an MRM pair equal to THCA-A, but was easily distinguished by retention time (3.9 vs 8.5 min, Fig 1D and 1F).

The isocratic LC conditions were developed to separate the target analytes from the family of congeners likely to be present in *C*. *sativa* extracts, and to generate the best peak shape. Formic acid was used to buffer the HPLC mobile phase to generate a single conjugate of the acids during chromatographic separation. The extraction procedure involved a large dilution, which was effective at extracting the analytes and diluting the matrix components sufficiently to negate matrix effects. The method is capable of analyte quantification at concentrations orders of magnitude below levels relevant for regulatory standards. The method was validated for additional dilutions in the instance of very high concentrations of the analytes in commercial products, making the range of concentrations facilitated by the method very wide.

### Method validation

Full method validation was conducted according to the FDA guideline using olive oil as matrix. Partial validation experiments were performed on two additional matrices, Delivra SR cream and dried plant material. Seven of the 8 oil matrices analyzed for selectivity contained no interfering substances with any of the analytes of interest or their internal standards. One brand of sunflower oil did contain a component that interfered with the analysis of CBD using this method. Representative chromatograms of blank matrix (A, B), the 4 cannabinoids and their internal standards spiked in blank matrix (C, D), and one test article (E, F) can be found in Fig 1.

LCMS run carryover was tested by injecting the blank matrix extraction (with IS) immediately after the analysis of the highest concentration (50 ng/mL) of the standard series. Lack of carryover was confirmed by a peak area of < 1/5 the LLOQ. Using this criteria, there was no carry over for the 4 analytes.

The calibration curve for 4 cannabinoids were constructed using a weighted quadratic curve of the peak area ratio of the analytes to their corresponding IS vs. concentration of analytes over the range of 0.195 ng/mL to 50 ng/mL. The limit of detection of CBD and THC was 0.048 ng/mL and CBDA and THCA-A was 0.024 ng/mL. Table 1 describes the linear range, correlation coefficients, LLOQ and LLOD for each analyte. The coefficient of variance for the LLOQ (0.19 ng/mL) was < 20%.

**Table 1:** Quantification range, correlation coefficient and LLOQ of calibration curves.

Analyte	Quantification Range (ng/mL)	Fitting Equation		Correlation Coefficient (R^2^)	LLOD (ng/mL)
CBD	0.195–50 (Linear)	y = 0.0731x+0.00922		0.9996	0.048
THC	0.195–50 (Linear)	y = 0.0949x+0.00393		0.9999	0.048
CBDA	0.195–50 (Quadratic)	y = -0.00713x^2^+2.1x+0.0638		0.9996	0.024
THCA-A	0.195–50 (Quadratic)	y = -0.0054x^2^+1.71x+0.0701		0.9999	0.024

Quality control samples in olive oil were analyzed on three days (n = 5) at concentrations from 0.5 ng/mL to 200 ng/mL. Table 2 describes intra- and inter-day precision and accuracy of the 4 cannabinoids in olive oil. Accuracies between 94.2–112.7% and 97.2–110.9% for intra- and inter-day were observed. Intra-day and inter-day precision of the 4 analytes determined at each concentration did not exceed 8.1% and 12.4% RSD respectively.

**Table 2:** Precision and accuracy of the determination of cannabinoids in extra virgin olive oil (Inter-day n = 5x3; Intra-day n = 5).

Analyte	Nominal Concentration (ng/mL)	Measured Concentration (ng/mL) (mean +/- SEM)	Accuracy (%)	RSD (%)
THC				
Intra-day	0.5	0.49 +/- 0.01	97.7	2.20
	5	4.97 +/- 0.02	99.3	1.00
	50	54.36 +/- 0.35	108.7	3.45
	200*	221.70 +/- 2.76	110.9	2.78
Inter-day	0.5	0.49 +/- 0.01	98.9	5.76
	5	5.11 +/- 0.05	102.3	3.62
	50	53.95 +/- 0.34	107.9	2.45
	200*	213.53 +/- 2.66	106.8	4.82
CBD				
Intra-day	0.5	0.50 +/- 0.02	100.0	7.94
	5	4.95 +/- 0.06	99.0	2.62
	50	51.60 +/- 0.23	103.2	1.00
	200*	218.50 +/- 1.82	109.3	1.87
Inter-day	0.5	0.49 +/- 0.01	97.2	7.20
	5	5.01 +/- 0.05	100.2	4.09
	50	52.25 +/- 0.41	104.5	3.02
	200*	213.13 +/- 2.72	106.8	4.93
CBDA				
Intra-day	0.5	0.52 +/- 0.01	103.7	4.19
	5	5.18 +/- 0.05	103.7	2.05
	50	56.20 +/- 0.95	112.4	3.76
	200*	225.40 +/- 4.22	112.7	4.19
Inter-day	0.5	0.52 +/- 0.01	103.8	7.30
	5	5.07 +/- 0.09	101.3	6.57
	50	55.57 +/- 0.43	111.1	2.96
	200*	214.00 +/- 3.59	107.0	6.50
THCA-A				
Intra-day	0.5	0.49 +/- 0.01	98.6	4.17
	5	4.71 +/- 0.14	94.2	6.82
	50	49.82 +/- 1.80	99.6	8.09
	200*	216.30 +/- 3.62	108.2	3.74
Inter-day	0.5	0.52 +/- 0.01	103.2	5.28
	5	5.01 +/- 0.10	100.3	8.11
	50	51.65 +/- 1.65	103.3	12.40
	200*	217.03 +/- 2.56	108.5	4.57

* Quality control samples of 200 ng/mL were diluted 1/5 with extraction solvent before injection.

Quality control samples of analytes in plant material and cream were analyzed on a single day (n = 5) at concentrations from 0.5 ng/mL to 200 ng/mL. Tables 3 and 4 describe intra-day precision and accuracy of the 4 cannabinoids in these matrices. For plant material, accuracy ranges of 100.4–105.6% and 101.5–108.1% were observed for THC and CBD, respectively (Table 3). Similarly, cream matrix accuracies of 91.7–113.7% and 98.7–114.1% were observed for THC and CBD, respectively (Table 4). As well the coefficient of variance was below 13.2% for both THC and CBD in both matrices. Unlike oil, the plant and cream matrix accuracies for CBDA and THCA-A were outside the acceptable range for FDA validation for both matrices and coefficient of variance also failed for both acids in either one or both matrices (Tables 3 and 4). Taken together, the method in its current state functions to accurately quantify all four cannabinoids in oil-based matrices and THC and CBD in a variety of matrices, however the accuracy of CBDA and THCA-A in complex plant materials and creams incorporates an increased level of uncertainty.

**Table 3:** Precision and accuracy of the determination of cannabinoids in plant material (Intra-day n = 5).

Analyte	Nominal Concentration (ng/mL)	Measured Concentration (ng/mL) (mean +/- SEM)	Accuracy (%)	RSD (%)
THC				
Intra-day	0.5	0.50 +/- 0.03	100.4	13.20
	5	5.23 +/- 0.07	104.5	3.42
	50	52.82 +/- 0.67	105.6	3.08
	200*	209.67 +/- 3.83	104.8	4.47
CBD				
Intra-day	0.5	0.52 +/- 0.02	103.9	11.51
	5	5.11 +/- 0.10	102.3	4.61
	50	50.77 +/- 0.20	101.5	0.95
	200*	216.24 +/- 3.01	108.1	3.41
CBDA				
Intra-day	0.5	0.91 +/- 0.09	182.3	25.43
	5	6.87 +/- 0.15	137.4	5.38
	50	78.98 +/- 4.35	158.0	13.50
	200*	229.47 +/- 6.90	114.7	7.38
THCA-A				
Intra-day	0.5	0.73 +/- 0.02	145.3	6.00
	5	7.25 +/- 0.35	145.0	11.98
	50	103.17 +/- 3.79	206.3	9.00
	200*	287.33 +/- 8.50	143.7	7.24

* Quality control samples of 200 ng/mL were diluted 1/10 with extraction solvent before injection.

**Table 4:** Precision and accuracy of the determination of cannabinoids in cream (Intra-day n = 5).

Analyte	Nominal Concentration (ng/mL)	Measured Concentration (ng/mL) (mean +/- SEM)	Accuracy (%)	RSD (%)
THC				
Intra-day	0.5	0.46 +/- 0.01	91.7	7.94
	5	5.01 +/- 0.04	100.2	1.86
	50	55.13 +/- 0.66	110.3	2.94
	200*	227.50 +/- 7.21	113.7	7.21
CBD				
Intra-day	0.5	0.51 +/- 0.03	102.9	12.77
	5	4.93 +/- 0.06	98.7	3.17
	50	57.03 +/- 0.86	114.1	3.69
	200*	225.60 +/- 5.16	112.8	5.60
CBDA				
Intra-day	0.5	0.43 +/- 0.01	85.5	4.05
	5	4.65 +/- 0.04	93.1	1.97
	50	31.25 +/- 0.89	62.5	6.97
	200*	211.83 +/- 0.58	105.9	6.69
THCA-A				
Intra-day	0.5	0.27 +/- 0.02	53.0	14.05
	5	3.37 +/- 0.18	67.4	13.31
	50	38.35 +/- 2.40	76.7	15.35
	200*	188.60 +/- 21.20	94.3	27.53

* Quality control samples of 200 ng/mL were diluted 1/10 with extraction solvent before injection

Extraction recoveries for THC, CBD, CBDA, and THCA-A from olive oil were between 87.5–98.5%, 86.9–109.6%, 91.6–100.0%, and 83.7–102.3%, indicating a satisfactory extraction procedure (Table 5). Matrix effects of THC, CBD, CBDA and THCA-A were 110.4–116.0%, 105.4–112.2%, 96.3–117.8%, and 92.7–107.8%, therefore no significant matrix effect in oil was observed.

**Table 5:** Extraction recovery, matrix effect of 4 cannabinoids (n = 6) and 3 internal standards (n = 24) in Extra Virgin Olive Oil (mean +/- SEM).

Extraction Recovery	**Nominal Concentration**	**THC (%)**	**THC-d3 (%)**	**CBD (%)**	**CBD-d3 (%)**	**CBDA (%)**	**THCA-A (%)**	**THCCOOH-d3 (%)**
	0.5 ng/mL	98.5 +/- 2.7	91.2 +/- 1.2	109.6 +/- 3.3	93.7 +/- 1.3	100.0 +/- 3.2	102.3 +/- 1.8	95.4 +/- 2.6
	5 ng/mL	87.5 +/- 2.0		86.9 +/- 2.8		93.2 +/- 3.9	97.9 +/- 3.1	
	50 ng/mL	93.5 +/- 1.6		89.2 +/- 0.9		91.6 +/- 0.8	85.7 +/- 3.0	
	200 ng/mL	90.8 +/- 2.7		92.2 +/- 2.7		95.3 +/- 3.2	83.7 +/- 2.8	
Matrix effect	0.5 ng/mL	111.4 +/- 3.0	109.7 +/- 1.4	106.6 +/- 6.2	106.8 +/- 1.5	96.3 +/- 7.7	92.7 +/- 1.6	105.2 +/- 2.8
	5 ng/mL	110.4 +/- 3.2		105.4 +/- 2.4		117.8 +/- 3.7	96.9 +/- 4.0	
	50 ng/mL	115.4 +/- 1.2		112.2 +/- 1.6		115.8 +/- 1.1	103.2 +/- 1.5	
	200 ng/mL	116.0 +/- 1.2		112.2 +/- 1.3		109.1 +/- 1.3	107.8 +/- 1.7	

Extraction recoveries of THC and CBD were 102.0–112.8% and 98.3–114.6% from plant material and 85.1–90.0% and 86.8–94.1% from Delivra SR cream, supporting a satisfactory extraction procedure (Tables 6 and 7). Likewise matrix effects were acceptable for THC and CBD at 102.0–112.8 and 91.2–129.4% for plant material and 79.4–93.1% and 83.8–100.2% for cream. Extraction efficiencies for the acids from plant material were acceptable between 94.9–106.6% and 99.8–112.5% for CBDA and THCA-A respectively, however deviated below acceptable levels when extracted from the cream matrix (Table 6). Matrix effects were more pronounced for CBDA in plant materials, with an enhancing effect particularly obvious at low concentrations (Table 6). The differences in the extraction and matrix effects between the acids and their internal standard combine to give an overall accuracy that is not within 15% of the desired value in all control samples (Table 6).

**Table 6:** Extraction recovery, matrix effect of 4 cannabinoids (n = 6) and internal standards (n = 24) in plant material and cream matrices (mean +/- SEM).

Extraction Recovery -Plant material	**Nominal Concentration**	**THC (%)**		**THC-d3 (%)**	**CBD (%)**	**CBD-d3 (%)**	**CBDA (%)**	**THCA-A (%)**	**THCCOOH-d3 (%)**
	0.5 ng/mL	108.3 +/- 6.4		115.4 +/- 0.7	113.9 +/- 4.4	113.4 +/- 1.4	106.6 +/- 5.2	112.5+/- 5.1	86.5 +/- 1.4
	5 ng/mL	110.6 +/- 0.8			107.1 +/- 1.7		98.2 +/- 2.6	108.7 +/- 4.6	
	50 ng/mL	112.8+/- 1.8			114.6 /- 3.9		97.4 +/- 2.4	108.2 +/- 10.4	
	200 ng/mL	102.0 +/- 1.7			98.3 +/- 1.8		94.9 +/- 1.9	99.8 +/- 1.8	
Matrix Effect -Plant Material	0.5 ng/mL	109.8 +/- 2.4		101.1 +/- 0.8	129.4 +/- 5.0	93.5 +/- 0.9	339.7 +/- 15.6	93.0 +/- 6.0	102.4 +/- 1.3
	5 ng/mL	110.6 +/- 0.8			98.9 +/- 1.6		129.7 +/- 1.8	96.0 +/- 2.0	
	50 ng/mL	112.8 +/- 1.8			91.2 +/- 1.1		119.8 +/- 5.9	87.9 +/- 2.2	
	200 ng/mL	102.0 +/- 1.7			100.5 +/- 1.3		111.2 +/- 1.8	116.7 +/- 1.8	
Extraction Efficiency—Cream	0.5 ng/mL	88.5 +/- 5.8		94.4 +/- 0.7	91.7 +/- 4. 8	95.3 +/- 0.7	74.6 +/- 1.3	53.7 +/- 3.2	94.4 +/- 0.7
	5 ng/mL	85.1 +/- 1.2			86.8 +/- 1.4		80.4 +/- 0.4	70.2 +/- 2.7	
	50 ng/mL	87.6 +/- 0.6			94.1 +/- 1.8		89.5 +/- 0.85	78.3 +/- 1.9	
	200 ng/mL	90.0 +/- 2.2			91.5 +/- 2.0		83.4 +/- 4.0	63.5 +/- 10.1	
Matrix Effect—Cream	0.5 ng/mL	81.2 +/- 3.3		84.0 +/- 1.3	100.2 +/- 5.1	95.3 +/- 1.1	101.0 +/- 1.8	101.8 +/- 7.2	84.0 +/- 1.3
	5 ng/mL	81.0 +/- 0.6			92.4 +/- 1.1		90.0 +/- 1.1	86.0 +/- 3.7	
	50 ng/mL	79.4 +/- 1.3			83.8 +/- 1.3		98.4 +/- 0.5	91.4 +/- 3.0	
	200 ng/mL	93.1 +/- 0.6			93.4 +/- 1.3		92.6 +/- 1.8	91.0 +/- 2.6	

Table 7 describes the stability of CBD, THC, CBDA and THCA-A under normal sample processing conditions; in the extraction solvent with olive oil matrix on the benchtop and as a processed sample in the autosampler. All analytes were stable in extraction solvent on the benchtop (20°C) for 12 h, in the autosampler (4°C) for 3 days.

**Table 7:** Stability of cannabinoids (n = 5), (mean +/- SEM).

	Benchtop Stability (12 h) (%)				Autosampler Stability (3 days) (%)			
	0.5 ng/mL	5 ng/mL	50 ng/mL	200 ng/mL	0.5 ng/mL	5 ng/mL	50 ng/mL	200 ng/mL
THC	96.5 +/- 2.4	100.0 +/- 0.6	100.2 +/- 0.5	102.8 +/- 1.1	96.9 +/- 2.5	100.1 +/- 0.8	99.9 +/-0.6	98.6 +/- 0.6
CBD	94.8 +/- 4.8	96.6 +/- 4.2	98.5 +/- 1.0	102.3 +/- 0.9	101.3 +/- 4.3	99.9 +/- 1.4	98.1 +/-0.8	98.0 +/- 0.5
CBDA	102.5 +/- 3.1	104.3 +/- 2.7	99.5 +/- 2.3	98.6 +/- 1.4	107.7 +/- 3.7	111.5 +/- 4.2	105.9 +/-1.4	99.8 +/- 1.6
THCA-A	90.6 +/- 4.2	96.6 +/- 7.9	92.6 +/- 5.4	82.5 +/- 3.3	99.7 +/- 1.7	114.6 +/- 3.3	101.0 +/- 1.5	96.4 +/- 1.7

Incurred sample reanalysis was performed on one randomly selected sample from each batch and reanalyzed after >1 month. Samples 20, 21, 24, and 33 were reanalyzed and variation values [(repeated concentration–initial concentration)/average concentration*100] ranged from -0.73 ~ -10.88%, -3.49 ~ 3.85%, -12.97 ~ 13.95%, and 0 ~ 13.19% for CBD, THC, CBDA, and THCA-A. The results support the reliability of the method and the stability of the 4 analytes in storage at 4°C for one month.

### Cannabinoids in commercial products

**Fig 2:**
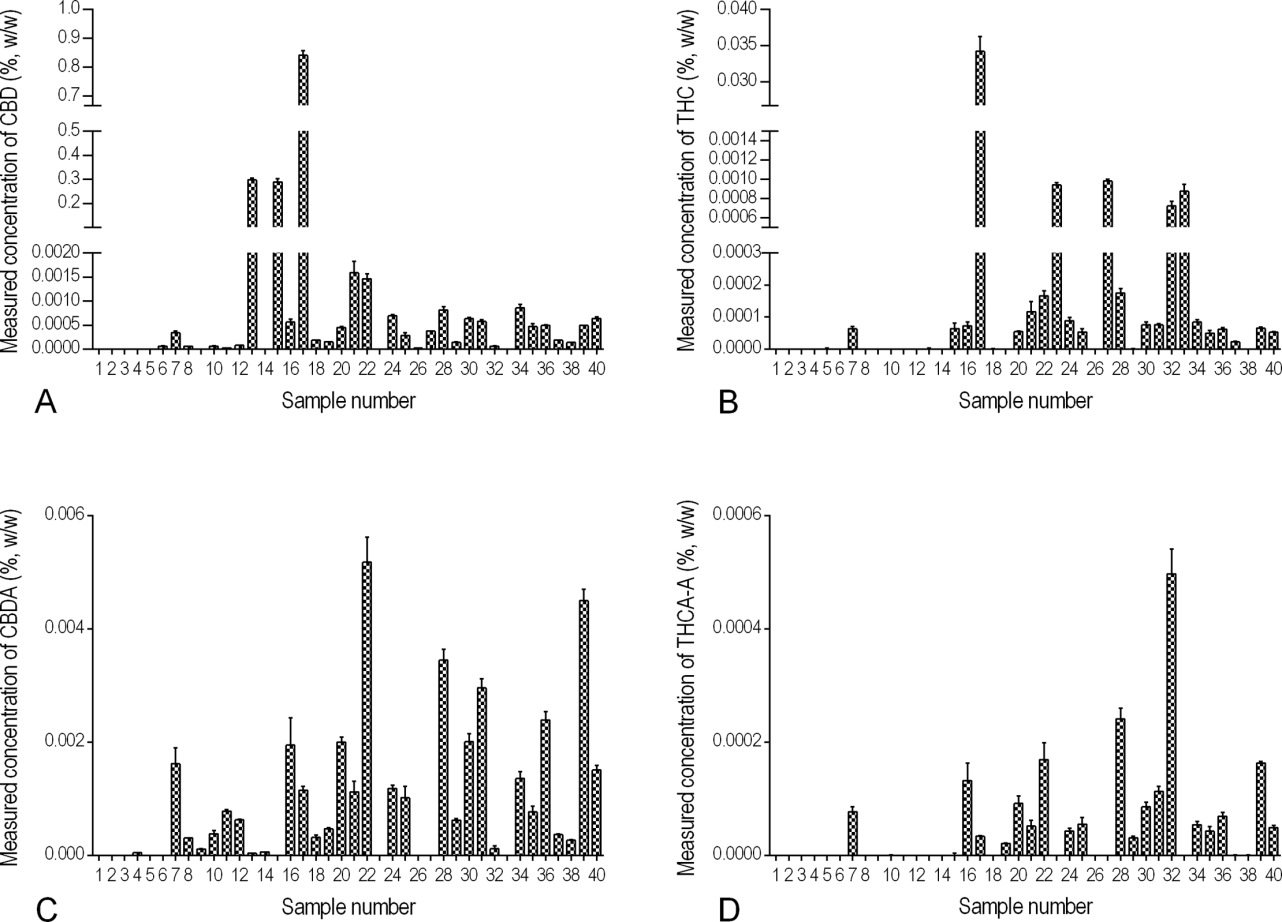
Comparative concentrations of cannabinoids in 40 commercial hemp finished products. CBD, THC, CBDA and THCA-A were extracted from test articles and quantified using LC-MS/MS. Measured levels, reported in % w/w of (A) CBD, (B) THC, (C) CBDA, and (D) THCA-A.

The validated method was applied to 40 finished products labelled as hemp-containing and available in Canada, ordered online or purchased in-store. The test articles included a variety of oils, plant materials, and creams in the forms of cosmetics, personal care products, hemp oils marketed for cooking, massage oil, body butter, tinctures, and supplements (Fig 2 and S1 Table). Within the 40 products tested, 38 products had THC levels well below the Canadian legal limit of 10 μg/g, one hemp oil (10.01 μg/g) contained slightly above that limit, and only one tincture (337 μg/g) was tremendously higher. That suggested the current regulations are working to limit access to high-THC *C*. *sativa* products marketed as hemp. The hemp products had a wide range of measured CBD concentrations, from below the limit of quantification (< 0.19 μg/g) to 8,410 μg/g. The product with the highest measured THC and CBD content contained 337 μg/g and 8,410 μg/g respectively (Fig 2, product # 17). The oil-based products were found to have overall higher levels of CBD and CBDA than the non-oil products in general. Most of the cosmetic products did not contain cannabinoid levels above the very low LLOQ, although they were marketed as hemp-containing. There was a large difference in the CBD content of the three products with the highest CBD content (Fig 2, products # 13, 15, and 17; >2,800 μg/g) and the remaining products (<20 μg/g). An interesting trend emerged between the concentration of CBD in a commercial sample and the ratio of the CBD:CBDA within the same sample. 90% of CBD and CBDA containing products had higher concentrations of CBDA than CBD. In stark contrast, the three products with the highest concentrations of CBD had very low relative levels of CBDA with CBD:CBDA ratios between 1,000:1.4 and 1,000,000:1.7. The ratio of THC:THCA or CBD:CBDA can vary in plants from strain to strain, but the lack of CBDA in the CBD-rich samples suggests an adulteration in the sample, either through a significant difference in processing methods or the addition of pure CBD to the end product.

Many nations stipulate a zero tolerance policy or maximum THC content in finished hemp products of 10 μg/g [34]. Medical doses of THC are generally described in increments of 10 mg [35] or 2.7 mg/dose for regulated drug products such as Sativex [36]. In contrast there is no defined acceptable CBD level or threshold for non-medical *C*. *sativa* agriculture [37]. This is partly fueled by a lack of required testing and the general safety of CBD, with case reports of safe consumptions levels of 3–1,500 mg/day and various clinical trials using 300–600 mg/day purport equivalent or lower side effects as compared to placebo [38,39]. Although further safety studies are required, the consensus is that CBD has no deleterious effects in humans, despite it being listed as a controlled substance in many countries. However, this compound is rarely encountered alone and the psychological outcome of THC consumption is moderated by the co-administration of CBD which commonly yields a lower intensity [40]. This alteration of THC’s action by CBD has been documented sufficiently to underline a need for all *C*. *sativa*-derived materials destined for human consumption to be tested for both analytes, rather than THC alone [41]. This becomes particularly relevant in recreational edibles, if consumed with hemp-derived oils, potentially modifying the final psychological outcome. Furthermore, patients receiving medical CBD within investigative studies or an eventual regulated drug (e.g. Epidiolex) must be provided with sufficient consumer knowledge to avoid unintentionally modifying their prescribed dosing. Lastly, it is of scientific and manufacturing benefit to evaluate THC, CBD, and their respective acids individually rather than converting the latter during the quantification process, as is common for GC-MS. While it is true that additive values (e.g. THC+THCA-A) represent the total potential dose, the consumer experience and concept of efficacy can be widely variable given that THCA-A itself has no neurological effects nor is its conversion to THC *in vivo* well understood [42,43].

## Conclusion

A sensitive, simple and fast method for the simultaneous quantification of THC, CBD, THCA-A and CBDA was developed and validated for testing oil-based products along with partial validation for these same compounds in plant material and cream-bases. Regarding the latter, acceptable validation was achieved for THC and CBD in plant material and cream demonstrating the broad utility of the method. Matrix challenges for the acid variants (THCA-A & CBDA) highlight that some internal standards fail in rigorous testing, and isotopic standards are preferable. To demonstrate the methods total utility, it was applied to 40 commercially available products. This method involved a simple extraction (10 mins) and rapid HPLC separation (11 mins) indicating a potential for high throughput and is applicable to numerous matrices. 38 of the commercial products had THC levels well below the Canadian legal limit of 10 μg/g and the method identified two products above this level. CBD concentrations from below the limit of quantification (< 0.19 μg/g) to 8,410 μg/g were observed. Given the high CBD content found in some products, it would be prudent for standard hemp testing to include both CBD and THC. In addition, the ratio of CBD:CBDA and THC:THCA may be valuable as a diagnostic tool of potential adulteration.

## Supporting information

S1 Table Cannabinoid content in consumer products. CBD, THC, CBDA, THCA content (μg/g) (mean+/- SEM, n = 3).
(PDF)
