# Supplementary material for: A reliable and validated LC-MS/MS method for the simultaneous quantification of 4 cannabinoids in 40 consumer products
Source: PLoS One. 2018 May 2;13(5):e0196396. doi: 10.1371/journal.pone.0196396 (PMC5931681; doi:10.1371/journal.pone.0196396)
Supplement: S1 Table — CBD, THC, CBDA, THCA content (μg/g) (mean+/- SEM, n = 3). (PDF) [file pone.0196396.s001.pdf]

Supplementary information

Table S1. Cannabinoids profile of consumer products. CBD, THC, CBDA, THCA content (mean +/- SEM, n=3) (µg/g) in commercial hemp products

Product Code	Form	CBD Present (µg/g)	THC Present (µg/g)	CBDA Present (µg/g)	THCA-A Present (µg/g)
1	Cream	BLQ	BLQ	BLQ	BLQ
2	Cream	BLQ	BLQ	BLQ	BLQ
3	Cosmetic	BLQ	BLQ	BLQ	BLQ
4	Cream	BLQ	BLQ	0.47 +/- 0.03	BLQ
5	Cream	BLQ	BLQ	BLQ	BLQ
6	Cream	0.64 +/- 0.02	BLQ	BLQ	BLQ
7	Oil	3.38 +/- 0.40	0.63 +/- 0.08	16.20 +/- 2.80	0.77+/- 0.09
8	Dried plant material	0.64 +/- 0.00	BLQ	3.08 +/- 0.01	BLQ
9	Dried plant material	BLQ	BLQ	1.14 +/- 0.10	BLQ
10	Seeds	0.60 +/- 0.10	BLQ	3.83 +/- 0.54	BLQ
11	Dried plant material - powder	0.27 +/- 0.02	BLQ	7.84 +/- 0.25	BLQ
12	Dried plant material - powder	0.79 +/- 0.03	BLQ	6.29 +/- 0.12	BLQ
13	Cosmetic	2,970 +/- 90	BLQ	0.39 +/- 0.02	BLQ
14	Oil	BLQ	BLQ	0.633 +/- 0.01	BLQ

15	Oil	2,890 +/- 130	0.64 +/- 0.17	BLQ	BLQ
16	Oil	5.60 +/- 0.63	0.73 +/- 0.12	19.5 +/- 4.8	1.32 +/- 0.31
17	Oil	8,410 +/- 160	337 +/- 21	11.5 +/- 0.74	0.34 +/- 0.02
18	Cream	1.83 +/- 0.07	BLQ	3.20 +/- 0.42	BLQ
19	Oil	1.49 +/- 0.02	BLQ	4.68 +/- 0.25	0.21 +/- 0.01
20	Oil	4.49 +/- 0.27	0.54 +/- 0.03	20 +/- 0.91	0.92 +/- 0.13
21	Oil	15.8 +/- 2.4	1.17 +/- 0.31	11.2 +/- 1.9	0.52 +/- 0.10
22	Oil	14.6 +/- 1.00	1.67 +/- 0.16	51.8 +/- 4.4	1.69 +/- 0.30
23	Capsule	BLQ	9.87 +/- 0.25	BLQ	BLQ
24	Oil	6.89 +/- 0.34	0.91 +/- 0.10	11.80 +/- 0.62	0.43 +/- 0.05
25	Oil	2.87 +/- 0.51	0.53 +/- 0.11	10.2 +/- 2.00	0.55 +/- 0.12
26	Oil	0.23 +/- 0.03	BLQ	BLQ	BLQ
27	Oil	3.71 +/- 0.07	10.01 +/- 0.19	BLQ	BLQ
28	Oil	8.16 +/- 0.67	1.78 +/- 0.13	34.50 +/- 1.90	2.41 +/- 0.19

29	Oil	1.41 +/- 0.09	BLQ	6.20 +/- 0.34	0.31 +/- 0.03
30	Oil	6.33 +/- 0.25	0.76 +/- 0.10	20.10 +/- 1.40	0.86 +/- 0.08
31	Oil	5.76 +/- 0.30	0.76 +/- 0.05	29.6 +/- 1.605	1.13 +/- 0.09
32	Oil	0.64 +/- 0.01	7.37 +/- 0.53	1.20 +/- 0.45	4.97 +/- 0.44
33	Capsule	BLQ	8.95 +/- 0.71	BLQ	BLQ
34	Capsule	8.58 +/- 0.77	0.85 +/- 0.07	13.6 +/- 1.20	0.54 +/- 0.06
35	Oil	4.70 +/- 0.55	0.49 +/- 0.08	7.68 +/- 0.98	0.43 +/- 0.08
36	Oil	4.89 +/- 0.17	0.62 +/- 0.05	23.90 +/- 1.50	0.69 +/- 0.07
37	Gel	1.83 +/- 0.02	0.20 +/- 0.02	3.67 +/- 0.17	BLQ
38	Gel	1.39 +/- 0.04	BLQ	2.66 +/- 0.10	BLQ
39-1	Oil	4.89 +/- 0.04	0.63 +/- 0.02	45.03 +/- 0.02	1.63 +/- 0.03
39-2	Oil	6.38 +/- 0.33	0.52 +/- 0.02	15.10 +/- 0.85	0.49 +/- 0.04

BLQ Below the lowest quantification limit (0.195 µg/g) in consumer products
